# An investigation of the influence of microstructure surface topography on the imaging mechanism to explore super-resolution microstructure

**DOI:** 10.1038/s41598-022-17209-9

**Published:** 2022-08-11

**Authors:** Wenpeng Fu, Chenyang Zhao, Wen Xue, Changlin Li

**Affiliations:** School of Mechanical Engineering and Automation, Harbin Institute of Technology, Shenzhen, 518055 China

**Keywords:** Mechanical engineering, Techniques and instrumentation

## Abstract

Vision-based precision measurement is limited by the optical resolution. Although various super-resolution algorithms have been developed, measurement precision and accuracy are difficult to guarantee. To achieve nanoscale resolution measurement, a super-resolution microstructure concept is proposed which is based on the idea of a strong mathematical mapping relationship that may exist between microstructure surface topography features and the corresponding image pixel intensities. In this work, a series of microgrooves are ultra-precision machined and their surface topographies and images are measured. A mapping relationship model is established to analyze the effect of the microgroove surface topography on the imaging mechanism. The results show that the surface roughness and surface defects of the microgroove have significant effects on predicting the imaging mechanism. The optimized machining parameters are determined afterward. This paper demonstrates a feasible and valuable work to support the design and manufacture super-resolution microstructure which has essential applications in precision positioning measurement.

## Introduction

Super-resolution (SR), which refers to the process of improving the resolution of original images by means of reconstructing high-resolution (HR) images from low-resolution (LR) images^1^, is widely used in microscopic imaging^2–4^, video surveillance^5^, medical imaging^6^, satellite remote sensing imaging^7^ and astronomical observation^8^, etc. Besides, SR methods also have essential applications in precision positioning measurement, and plays an important role in the improvement of positioning accuracy^9,10^. Normally, micro-vision-based precision positioning measurement methods^11–15^ improve the resolution mainly by using image processing methods^11,15^. When the similarity of certain image areas is high, algorithms easily cause matching errors, thus seriously decreasing the measurement accuracy and uncertainty.

Currently, SR reconstruction of images is mainly achieved from the perspective of software algorithms, such as the Deep Plug-and-Play Super-Resolution (DPSR) algorithm^16^, unpaired image confrontation network^17^ for generalization ability, feature map attention mechanism to enhance the feature expression ability of reconstructed images^18^ and so on. But due to Abbe's limit, the resolution limit of ordinary optical microscopes is approximate to 200 nm. Hence, micro-topography information below the scale of 200 nm cannot be obtained by optical microscopes. Image SR reconstruction is not able to solve the loss of sampling high-frequency information of the observed object surface image at microscopic scale only from the viewpoint of algorithms. It is very challenging to break through the optical limit and realize the super-resolution imaging of microstructure surface topography.

## Super-resolution microstructure

**Figure 1:**
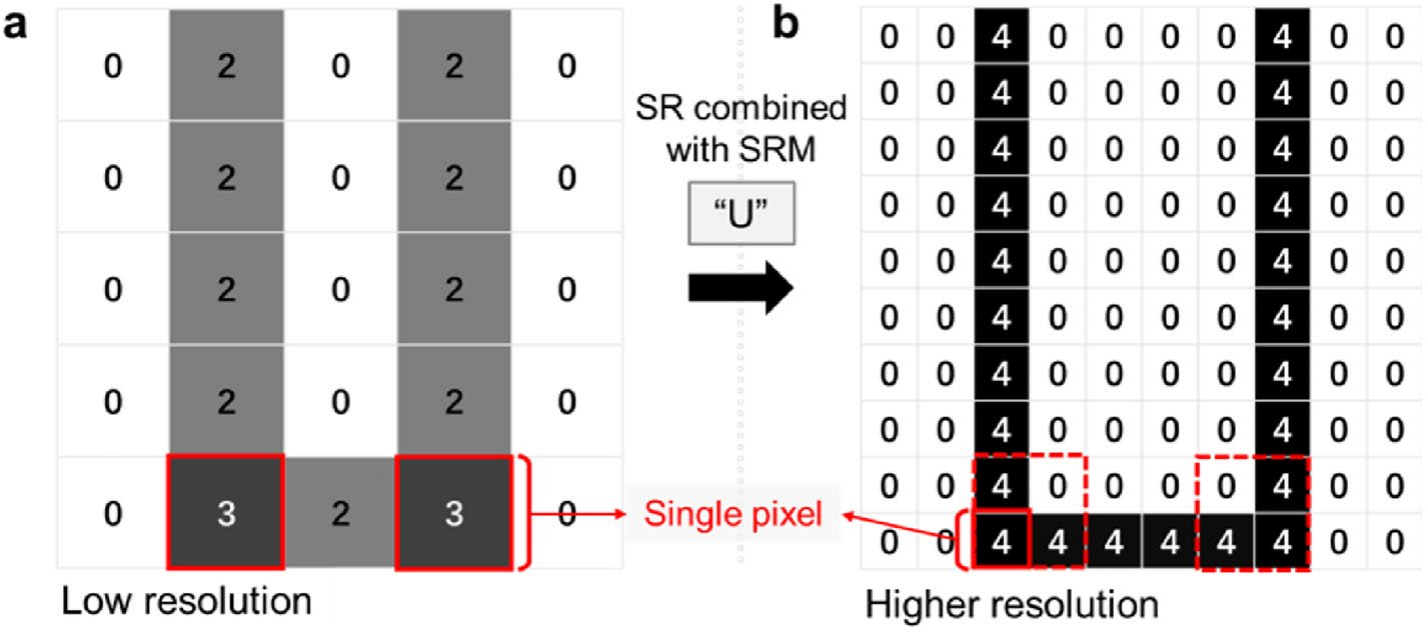
Super-resolution reconstruction based on SRM. (**a**) Low-resolution image; (**b**) Super-resolution reconstruction image combined with SRM "U".

Here, an innovative idea is generated: whether there is a micro-topography with SR characteristics, which is named "super-resolution microstructure" (SRM). Specifically, within the range of an individual pixel size as shown in Fig. 1a, although this area is extracted by the pixel data structure of only one pixel through a microscope, the original pixel can be decomposed into valuable sub-pixels that truly reflect the micro-topography characteristics through its neighboring pixel information as shown in Fig. 1b and the decoding characteristics of SRM, so as to realize SR.

As the SRM surface is observed, the image used for registration can have higher resolution and more reliable image details through feature function interpolation, so that the differences of details in each region are more obvious and easier to be identified stably by the algorithm, which can provide more accurate position feedback to measure the object and improve the positioning accuracy.

Therefore, the advantages of the SRM imaging method can be summarized as follows: (1) It can break through the optical limit from other perspectives and achieve reliable image super-resolution. (2) Compared with the traditional interpolation algorithms to achieve image super-resolution, it has a more dependable mathematical relationship. (3) Using SRM as the observation object of micro-vision-based precision positioning measurement, the imaging resolution is able to achieve the nanoscale level^13–15,19^.

However, it is quite challenging to realize SRM, which mainly comes down to: (1) Imaging principles and hardware conditions which limit image resolution and image effect; (2) In terms of processing mechanism, how to ensure the repeatability and expandability of microstructure surface topography; (3) Currently, there is little research on the correlation interpolation function proposed by exploring the influence of the microstructure surface topography distribution characteristics of the imaging mechanism. It is necessary and valuable to focus on mapping the relationship between the microstructure surface topography and image pixel intensity so as to explore the influence of microstructure surface topography on the imaging mechanism before designing and manufacturing SRM.

In this paper, in order to reveal the influence of surface topography on the imaging mechanism, experiments of microgroove surface topography imaging under different processing parameters are conducted. First, an ultra-precision machining experiment is carried out to generate microgrooves. A white light interferometer (WLI) is used to analyze the surface topography of the microgrooves. Then, the height data of microgroove topography and image pixel data with one-to-one correspondence are obtained. The correlation between the topography height data and the image pixel data, and the mapping relationship between the surface topography of the longitudinal section of the microgrooves and the corresponding image are analyzed at the nanoscale level. Finally, the relevant law of the influence of microgroove surface topography on the imaging mechanism is summarized, which is the basis for studying the sub-pixel interpolation algorithm for SRM.

## Experiment details

**Figure 2:**
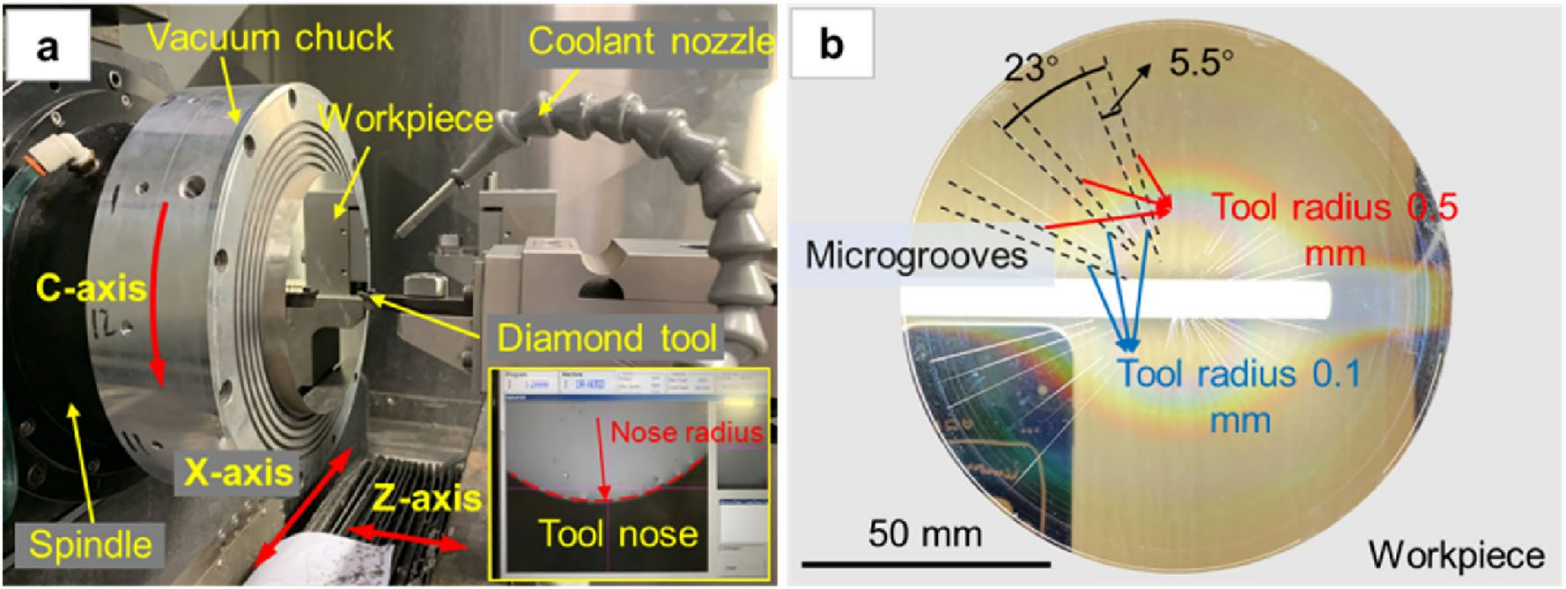
(**a**) Experiment setup, and (**b**) Machined workpiece.

An experiment involving the machining of microstructure surface was carried out on a three-axis (X-, Z- and C-axis) CNC ultra-precision single-point diamond lathe (Moore Nanotech 450 UPL, USA); the experiment setup is as shown in Fig. 2a. The lathe spindle motion accuracy is higher than 12.5 nm, the axes motion accuracy is 0.3 μm, and the programming resolution is 0.01 nm (linear)/0.000001 degree (rotary). Because the cupronickel is beneficial for maintaining the characteristics of the workpiece surface, and can improve the imaging stability of the microstructure surface, the substrate material of workpiece is chosen to be B15 cupronickel. The microstructure topography involved in this experiment is realized by diamond-cutting of the microgrooves. In order to control the variables, the whole workpiece surface was firstly pre-machined with a 0.5-mm tool nose radius to a level of surface roughness of 10 nm, and the spindle speed, feed rate and depth of cut amount were 1500 r/min, 5 mm/min and 8 μm respectively. Synthetic isoparaffins (Isopar fluids, ExxonMobil Chemical) were used as the coolant to improve the surface cutting quality. The tool nose radius used for microgroove cutting on the surface of the unit microstructure experiment workpiece was 0.5 mm and 0.1 mm respectively.

To study the influence of microstructure surface topography on the imaging mechanism, a series of microgroove cutting experiments was conducted with different cutting parameters. As shown in Table 1, five sets of cutting speeds (800, 400, 200, 100 and 50 mm/min) were set in the machining experiment, and the depth of cut was 5 μm. Repeat experiments were also conducted for each set of the same cutting speed. And microgrooves with three different lengths were cut at each cutting speed according to the microgroove length set in Table 1, in order to better distinguish each microgroove on the end face of the workpiece. The workpiece was clamped in the grooving process, the microgrooves under various parameters were cut at 23-degree intervals in the order of group number, and the offset angle of microgrooves was 5.5 degrees for the same group number. The microgroove spacing layout is shown in Fig. 2b.

**Table 1:** Experiment groups and machining parameters.

Group no	Cutting parameters		
	Cutting speed (mm/min)	Microgroove length (mm)	
		(Nose radius 0.5 mm)	(Nose radius 0.1 mm)
1–3	800	52, 51.1, 49.8	53.2, 51.8, 50.9
4–6	400	48.6, 47.3, 46.1	50, 48.9, 48
7–9	200	44.7, 43.5, 42.3	47.1, 46.1, 45.2
10–12	100	40.9, 39.7, 38.4	44.3, 43.3, 42.5
13–15	50	37.1, 35.9, 34.6	41.5, 40.5, 39.7

After the cutting experiment, a WLI (Bruker Contour GT-X) was used to measure the three-dimensional surface topography of the microstructure topography, and the point cloud coordinate dataset of the microstructure topography and the corresponding image pixel intensity dataset were acquired by the analysis software of the WLI.

## Results and discussion

### Qualitative analysis of the relationship between surface topography and image intensity distribution

**Figure 3:**
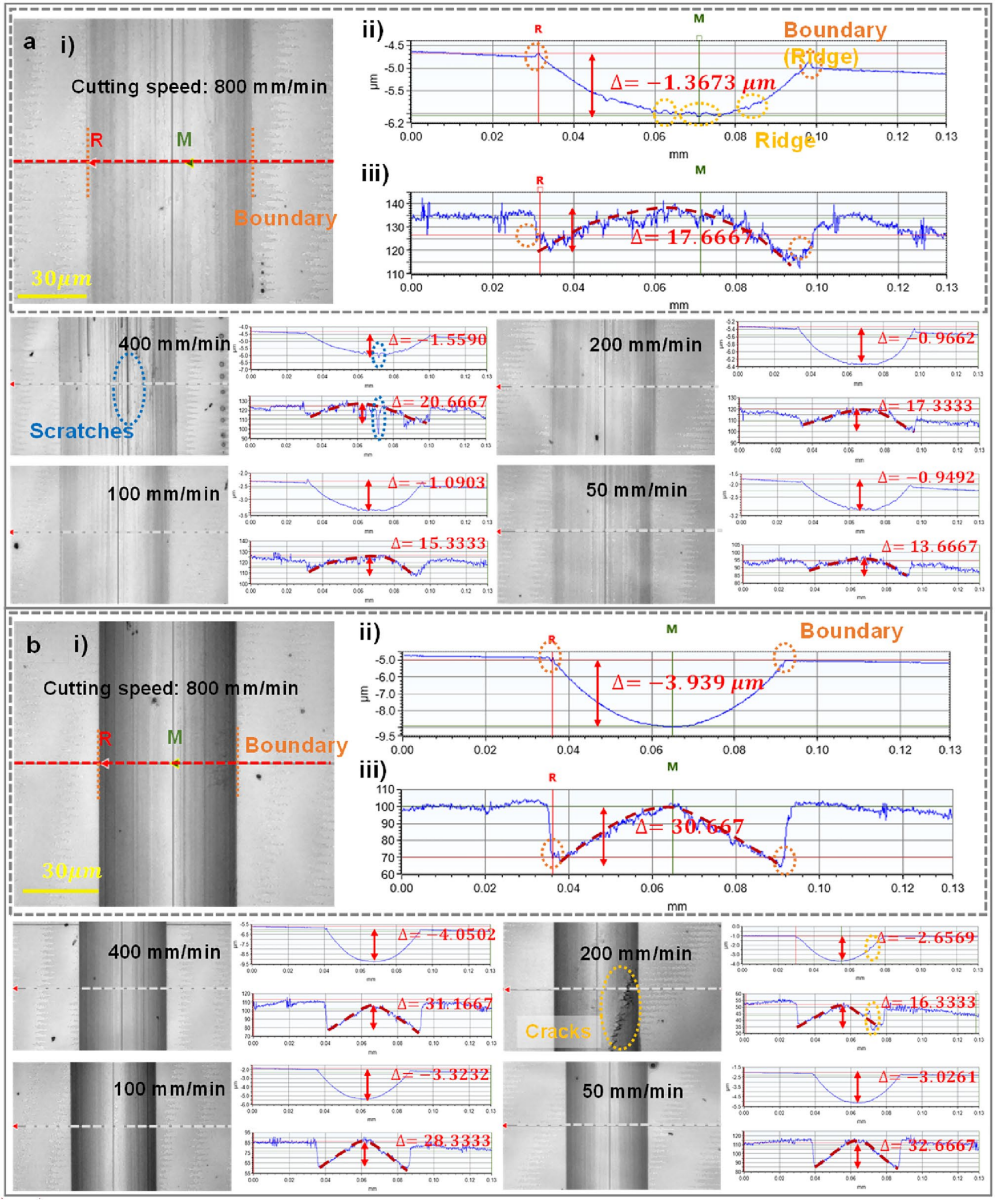
(**a**) Pixel intensity distribution of the microgroove image response to topography at different cutting speeds, with a nose radius of 0.5 mm; (i) grayscale image of microgrooves at a cutting speed of 800 mm/min, (ii) topography height distribution of the longitudinal section of the microgrooves, (iii) image pixel distribution of the longitudinal section of the microgrooves; (**b**) Pixel intensity distribution of the microgroove image response to topography at different cutting speeds, with a nose radius of 0.1 mm; (i) grayscale image of microgrooves at a cutting speed of 800 mm/min, (ii) topography height distribution of the longitudinal section of the microgrooves, (iii) image pixel distribution of the longitudinal section of the microgrooves.

To understand the influence of the microstructure surface topography on the imaging mechanism, it was necessary to analyze the response mechanism of the image pixel intensity distribution in relation to the microstructure topography. The repeat experiments set three different lengths of microgrooves for each cutting parameter. From the microgroove topography results collected by the WLI, under the same cutting parameters, the cutting length had little effect on the microgroove topography distribution, so the data of one micro-groove was selected under each cutting parameter for comparative analysis. The effects of the surface topography under different cutting parameters on the imaging mechanisms of microgrooves are shown in Fig. 3. The experiment results show that the pixel intensity of the microgroove topography image underwent a sudden change at the boundary of the microgrooves, and the pixel intensity distribution in the area within the boundary was found to be negatively correlated with the topography height distribution, which was consistent with the visible light imaging mechanism. Due to the tool setting error and material removal characteristics during the machining process, the height difference of the topography under each parameter fluctuated within a certain range. In addition, the illumination conditions when operating the WLI to collect sample data affected the overall distribution interval of the pixel intensity of the micro-groove image. Therefore, the trend change of data distribution was used to characterize the response mechanism of the image pixel intensity distribution in relation to the microstructure topography. In general, with a decrease of cutting speed, the surface topography of microgrooves and the intensity distribution of image pixels do not change significantly. When the tool nose radius was 0.5 mm, the two boundaries and bottom of the microgroove produced obvious extrusion plastic deformation topography, and their image pixel intensity distribution produced the corresponding responses, as shown in Fig. 3aii,iii. When the tool nose radius was 0.1 mm, the surface processing quality of the microgrooves was better, and the change of the image pixel intensity distribution was relatively smooth, as shown in Fig. 3bii,iii. It is valuable to notice that under specific cutting parameters, the chip particles retained near the cutting edge of the tool were likely to rub against the machined surface during the grooving process, or the tool tip cut through the cracked part of the workpiece material, resulting in straight line scratches and defects such as cracks at the bottom of the microgroove. And the image pixel intensity distribution responds significantly to the surface topography of the cross-section when passing through these defects, as shown in Fig. 3a,b. To sum up the above, the pixel intensity distribution of the microgroove image has strong correlation with a change of topography, and the premise of obtaining a valuable image pixel intensity distribution law is that the surface quality of the fabricated microgroove is as good as possible.

### Correlation analysis

The geometries of the longitudinal sections of the microgrooves are similar, and the imaging mechanism of the microgrooves also exhibits similar laws. In order to better analyze the influence of microgroove topography on the imaging mechanism under different cutting parameters, the correlation between the topography height of the microgroove along the longitudinal section and the corresponding image pixel intensity was further studied quantitatively. Both the topography height coordinates and image pixel data sampled by the 3D topography instrument can be represented as a matrix with a horizontal resolution of 128 nm (objective magnification: × 50) and a dimension of 1376 × 1032. Therefore, there are 1,376 pairs of longitudinal section data samples under a set of cutting parameters, and the correlation coefficient “r” (absolute value) of each pair of data was calculated by Eq. (1):

1 $$r(X,Y) = \frac{{Cov(X,Y)}}{{\sqrt {Var[X]Var[Y]} }}$$

where *Cov(X,Y)* is the covariance of *X* and *Y*, *Var[X]* is the variance of *X*, and *Var[Y]* is the variance of *Y*.

**Figure 4:**
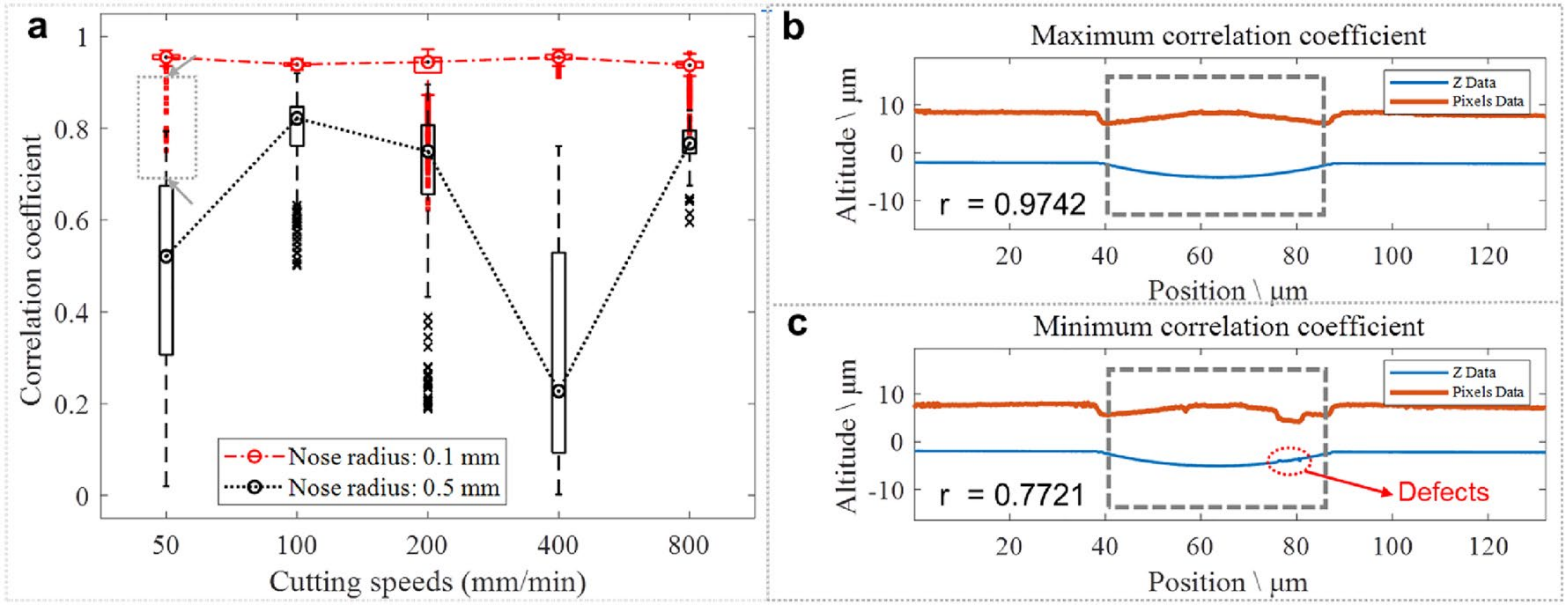
(**a**) Correlation coefficient of microgroove longitudinal section topography height and image pixel intensity distribution under different ultra-precision cutting parameters. (**b**) Distribution of longitudinal section data corresponding to the maximum correlation coefficients (the tool nose radius: 0.1 mm, and the cutting speed: 50 mm/min). (**c**) Distribution of longitudinal section data corresponding to the minimum correlation coefficients (the tool nose radius: 0.1 mm, and the cutting speed: 50 mm/min).

Figure 4 shows the correlation coefficient between the height distribution of the microgroove longitudinal section and the image pixel intensity distribution under different ultra-precision cutting parameters. As shown in Fig. 4a, when the nose radius is 0.5 mm, the cutting speed has a greater influence on the correlation, and the difference in the distribution of the correlation coefficient in the same group also reflects the instability of the imaging mechanism. Among them, the correlation coefficient of the cutting speed 100 mm/min group is generally higher than that of the other groups, and the correlation between the topography height of the microgroove and the image pixel intensity distribution is relatively significant. However, when the nose radius is 0.1 mm, except for the outlier correlation coefficient values corresponding to a small number of longitudinal sections, the height of the longitudinal section at several cutting speeds is significantly related to the pixel distribution of the image, with little difference in correlation degree, and the imaging mechanism is more regular. Especially when the cutting speed is 100 mm/min and the nose radius is 0.1 mm, the distribution of correlation coefficient is the most concentrated, and the imaging mechanism is highly consistent. In addition, the influence of defects on the imaging mechanism of microgroove topography is also reflected in the correlation coefficient. Combined with Fig. 3, the pixel intensity distribution of the image has a response to the local defects of the microgroove topography under different ultra-precision cutting parameters, and the correlation coefficient between the longitudinal section topography and the image data distribution passing through the local defects is smaller than that of other parts. For example, when the cutting conditions with the tool nose radius was 0.1 mm, the cutting speed was 50 mm/min, and the absolute value of the correlation coefficient “r” was between 0.7721 and 0.9742, the microgroove topography height had a significant correlation with the image pixel intensity distribution. At the longitudinal section positions where the correlation coefficients “r” were 0.9742 and 0.7721, respectively, the topography height and image pixel intensity distribution are shown in Fig. 4b,c. It can be seen from Fig. 4c that the defects have a significant impact on the imaging of the microgroove, manifesting in the fact that the machining or surface roughness has a great influence on the correlation. Therefore, the microgroove topography with fewer defects is beneficial to ensure the linear correlation between the surface topography height and the image pixel intensity.

### Mapping relationship model between surface topography and image intensity

#### Mapping relationship Model establishment

Quarrying a decoupling mathematical mapping relationship between the microstructure topography and the image pixel intensity is the key to realizing SRM but is difficult. In reality, precision measuring instruments such as the WLI can be used to obtain the nanometer-level resolution microstructure topography height point cloud coordinates and images, while the resolution of images obtained with general microscopic imaging is usually far below the nanometer level. Through the mapping relationship, if the nanoscale resolution topographic height data “Z” are obtained, the corresponding resolution image can also be obtained. Therefore, a mapping relationship model between the microgroove topography and its image is established here.

**Figure 5:**
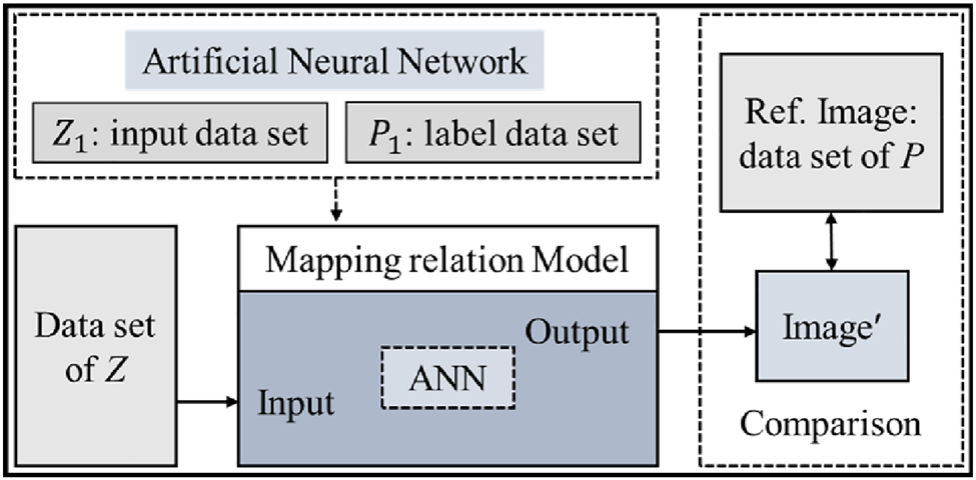
The architecture of the mapping model between microgroove topography and image.

As shown in Fig. 5, the microgroove longitudinal section topography height dataset “Z_1_” and image pixel intensity dataset “P_1_” are used as input value and label value respectively, and the mapping relationship model of them is iteratively calculated by a fully connected neural network. The reference image (Ref. image) pixel intensity dataset “P” is corresponding to the dataset “Z”. The Ref. image is compared with the output map image (“Image’”) using the dataset “Z” test the mapping relationship model to visualize and quantitatively evaluate the quality of the mapped image.

#### Mapping relationship model analysis under different cutting parameters

In order to analyze the mapping relationship between the microgroove topography and its image, a mapping relationship model under different cutting parameters was built. The model was trained using an artificial neural network (ANN, hidden layer size: 8; ratios of training, validation, and test sample data were 0.7, 0.2, and 0.1, respectively) based on the BP algorithm (Levenberg–Marquardt backpropagation). The computer used had an Intel(R) Core(TM) i9-7960X CPU @ 2.80 GHz 2.81 GHz with 64.0 GB of RAM.

**Figure 6:**
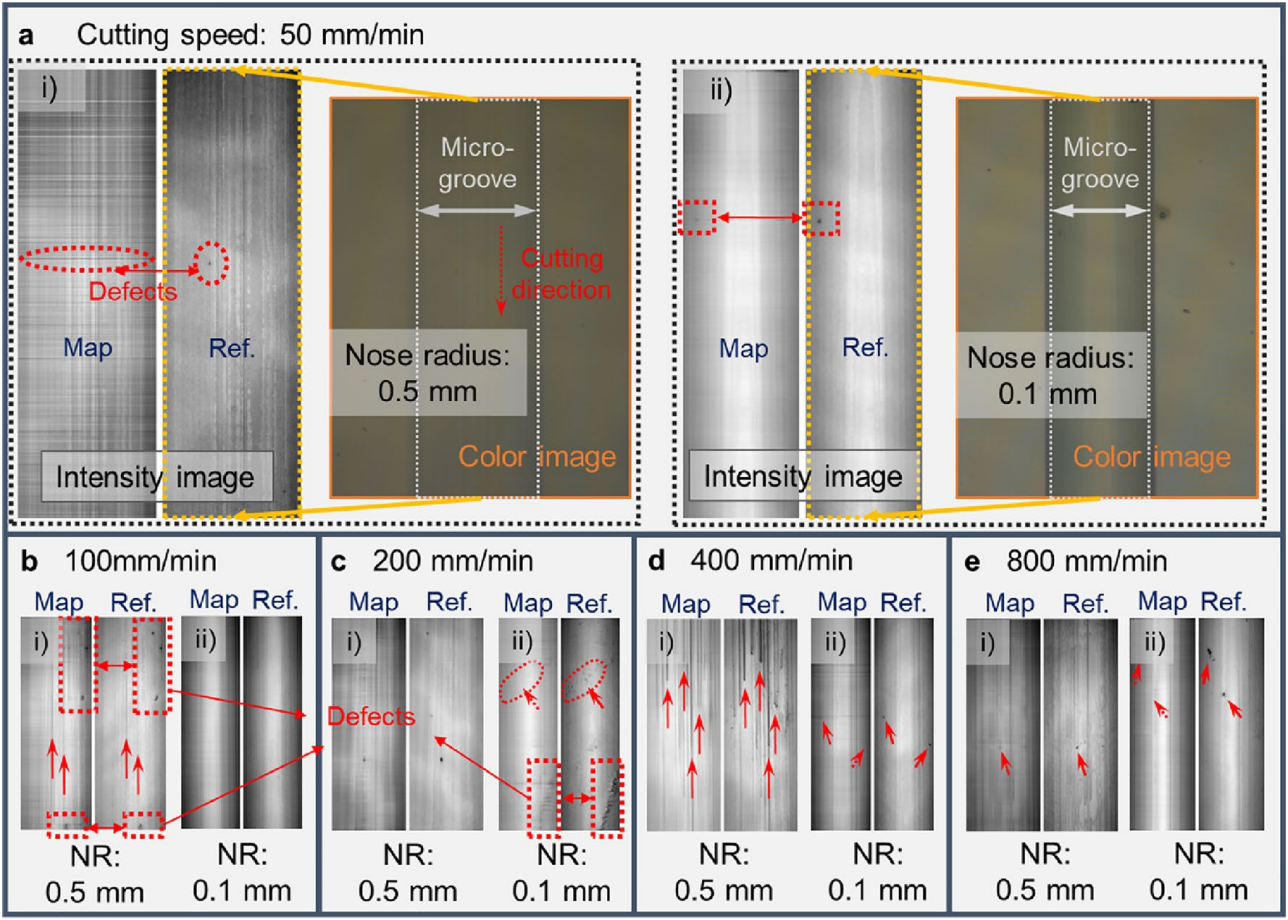
Comparison of microgroove topography mapped images and Ref. images under different cutting parameters. (**a**) Comparison of intensity images of microgrooves at a cutting speed of 50 mm/min; (i) tool nose radius (NR) of 0.5 mm, (ii) tool nose radius of 0.1 mm, and the mapped image is arranged next to the Ref. image (the same below). (**b**) Cutting speed of 100 mm/min. (**c**) Cutting speed of 200 mm/min. (**d**) Cutting speed of 400 mm/min. (**e**) Cutting speed of 800 mm/min.

The dataset of microgroove topography height in the test data under different cutting parameters was used as the input, and the mapped image was the output. The mapped image was compared with the Ref. image to analyze the effect of topography characteristics under different cutting parameters on the mapping relationship between the microgroove topography and the corresponding image. As shown in Fig. 6, the topographic features of microgrooves machined with different cutting parameters have different mapping imaging performances. From Fig. 6a, it is found that the texture distribution in the cutting direction of the microgroove topography mapped image corresponding to a tool nose radius of 0.1 mm at a cutting speed of 50 mm/min is closer to the Ref. image. In the mapped images with different cutting speeds, the surface defect features may cause disordered pixel intensity distribution in the mapped image as shown in Fig. 6ai, or be reproduced in the mapped image as shown in Fig. 6bi, or exhibit weaker pixel intensity in the mapped image as shown in Fig. 6aii,cii, or be lost in the mapped image as shown in Fig. 6cii, eii. On the whole, the regularity of the topography and texture features in the cutting direction is conducive to establishing a stable topography and image mapping relationship. However, the topography height distribution trend corresponding to the defect changes drastically, which has an unstable influence on the topography texture mapping. It is not only related to the topography distribution, but also has a relationship with the structure of the training model. Therefore, in the future, the processing of the mapping relationship of special topography features such as defects needs to be focused on.

**Figure 7:**
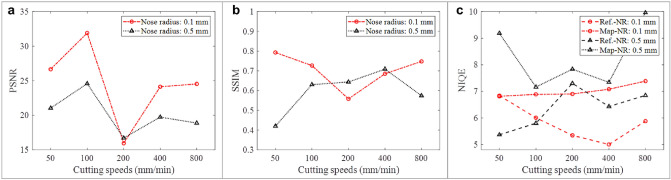
(**a**) PSNR comparison of mapped image under different cutting parameters; (**b**) SSIM comparison of mapped image under different cutting parameters; (**c**) NIQE comparison between the mapped image and the Ref. image under different cutting parameters.

Image quality metrics, such as Peak Signal-to-Noise Ratio (PSNR), Structural Similarity Index Measure (SSIM), and Natural Image Quality Evaluation (NIQE), were used to evaluate the quality of mapped images under different cutting parameters. As shown in Fig. 7a, with an increase of cutting speed, the PSNR values of the mapped images with a nose radius of 0.1 mm and 0.5 mm have similar trends. Especially at the cutting speed of 100 mm/min, the PSRN value reaches the maximum, which indicates that the overall pixel intensity values of the mapped image and the Ref. image are relatively close. However, at a cutting speed of 200 mm/min, the topography features and image pixel intensity are not well mapped, that is, the predictability of the image texture is poor. According to Fig. 6, under the same tool nose radius, compared with other cutting speeds, the texture and defect features in the Ref. image corresponding to 100 mm/min are relatively well reproduced in the mapped image, while the defect features in the Ref. image corresponding to 200 mm/min are not reproduced well, and these are reflected in the PSNR values.

From Fig. 7b, it can be seen that when the cutting speed is 50 mm/min and the nose radius is 0.1 mm, the SSIM value is the closest to 1. The distribution trend of the corresponding topography height of the longitudinal section of the microgroove is relatively stable, the mapped image has a high structural similarity with the Ref. image, and the topographical structure features of the microgroove and the image pixel intensity distribution characteristics are well mapped. But when the cutting speed is 50 mm/min and the tool nose radius is 0.5 mm, the SSIM value is the smallest. The corresponding microgroove topography roughness is relatively large, the structural similarity between the mapped image and the Ref. image is poor, and the correlation between the microgroove topographic structure feature and the image pixel intensity distribution feature is not strong. The perceptual quality of the mapped image with the Ref. image under different cutting parameters was compared, which is shown in Fig. 7c. From the mapped image, the NIQE value of the tool nose radius of 0.1 mm is smaller than that for 0.5 mm, which indicates better image perception quality. In addition, the perceptual quality of the mapped image is generally worse than that of the Ref. image, but when the cutting speed is 50 mm/min and the tool nose radius is 0.1 mm, the NIQE value of the mapped image is slightly smaller than that of the Ref. image, and the perceptual quality of the two is close. When the tool nose radius is 0.1 mm, the NIQE value of the mapped image tends to decrease narrowly with the decreasing cutting speed, and when the cutting speed is 50 mm/min, the NIQE value is the smallest, which indicates that the image perception quality is better. Therefore, comprehensively referring to the image quality indicators, it is concluded that the microstructure topography machined with a nose radius of 0.1 mm is more suitable for the study of the mapping relationship between the microgroove topography distribution and the image pixel intensity distribution, and a larger cutting speed can be selected to improve the cutting efficiency of the microstructure workpiece on the premise of ensuring the surface topography roughness.

Through the above analysis, under different ultra-precision cutting parameters, the distribution characteristics of microgroove surface topography largely affects the stability and the quality of the relationship between the microgroove topography and its mapped image. Therefore, selecting appropriate topography is the basis for ensuring the stable and decoupled mathematical mapping relationship between the microgroove topography distribution and the corresponding image pixel intensity distribution. For example, the topography corresponding to the cutting speed of 50 and 100 mm/min with a nose radius of 0.1 mm has fewer defects, a large correlation between the topography and the image, and a relatively stable mapping relationship, which is suitable for studying the sub-pixel interpolation algorithm for SRM.

## Conclusions

This paper experimentally provides an understanding of the influence of the microgroove surface topography on the imaging mechanism under different nose radius and cutting speed. The results show that the surface roughness and surface defects of micro-grooves have significant effects on the prediction of imaging mechanism, which is valuable to support the design and manufacture of SRM for micro-vision-based precision positioning measurement methods in the nano-scale. Considering the influence of illumination of the pixel intensity of microstructure imaging, nanoscale imaging experiments with controlled illumination will be performed to reveal the influence of the mapping relationship between the microstructure surface topography height distribution and the corresponding image intensity in future work.

## Data Availability

All data generated or analyzed during this study are included in this published article.
